# S100A8/S100A9 through PAD4 activation of neutrophil extracellular traps promotes granulomatous lobular mastitis

**DOI:** 10.3389/fimmu.2025.1672538

**Published:** 2025-12-16

**Authors:** Tingting Zhu, Hao Yu, Wei Wang, Yulu Sun, Shengjia Wang, Dongwen Ma, Yongzhong Yao

**Affiliations:** 1 Division of Breast Surgery, Department of General Surgery, Nanjing Drum Tower Hospital, the Affiliated Hospital of Medical School, Nanjing University, Nanjing, China; 2 Department of Breast Surgery, Xuzhou Central Hospital, The Affiliated Xuzhou Hospital of Medical College of Southeast University, Xuzhou, China

**Keywords:** granulomatous lobular mastitis, neutrophil extracellular traps, S100A8/S100A9, paquinimod, pad4

## Abstract

**Background:**

Granulomatous lobular mastitis (GLM) is a nonspecific chronic inflammatory breast disorder with an obscure etiology and pathogenesis. Neutrophil extracellular traps (NETs), which are extracellular web-like structures composed of decondensed chromatin and granular proteins released by activated neutrophils, disrupt normal tissue architecture and perpetuate inflammatory responses. The aim of the present study was to explore the role of NETs in GLM and the underlying regulatory mechanisms.

**Methods:**

Neutrophils were isolated from the blood of GLM patients and healthy controls (HCs) to assess NET formation. The presence of NETs in GLM tissues was detected using Western blot, immunohistochemistry, and immunofluorescence analyses. A mouse model of GLM was established to determine whether the inhibition of NET production, which is dependent on S100A8/S100A9, alleviates mammary gland inflammation. The potential mechanisms and therapeutic implications were further explored through *in vitro* and *in vivo* assays.

**Results:**

NETs were significantly increased in GLM tissues, as characterized by elevated levels of citrullinated histone H3 (CitH3) and myeloperoxidase (MPO). S100A8/S100A9 was highly expressed in GLM and demonstrated significant diagnostic value alongside NET markers. Mechanistically, S100A8/S100A9 promoted NETosis through interactions with peptidylarginine deiminase 4 (PAD4). Both paquinimod (an S100A8/S100A9 inhibitor) and Cl-amidine (a PAD4 inhibitor) effectively suppressed NET formation *in vitro*. In the GLM mouse model, both inhibitors reduced mammary gland inflammation, NET accumulation, and tissue damage.

**Conclusions:**

The present findings indicated that NETs contribute to the pathogenesis of GLM. S100A8/S100A9 plays a critical role in promoting NET formation via PAD4 activation. Targeting this axis with paquinimod effectively inhibits NETosis and alleviates GLM, suggesting a promising therapeutic strategy for GLM and other inflammatory diseases.

## Highlights

- Paquinimod inhibits NETs formation by modulating S100A8/S100A9.
- S100A8/S100A9 is an interventional target for inhibiting NETs in Granulomatous lobular mastitis.
- The present findings suggest the potential application of this pathway in the treatment of other inflammatory diseases.

## 1 Introduction

Granulomatous lobular mastitis (GLM) is a rare, benign and chronic inflammatory lesion of the breast (1). GLM is typically characterized by lobular noncaseating granulomas composed of epithelioid cells and multinucleated giant cells. Additionally, a predominantly neutrophilic infiltrate exists within and around the granuloma (2). Studies have suggested that certain factors, such as α-1 antitrypsin deficiency, cigarette smoking, Corynebacterium infections, pregnancy, breastfeeding, hyperprolactinemia, oral contraceptives, and autoimmune abnormalities, may be associated with the development of GLM (3–5). Recent studies have suggested that granulomatous lobular mastitis is likely an autoimmune disease (6–8). However, the precise etiology and pathogenesis of GLM remain incompletely understood.

Neutrophils are the most abundant type of leukocyte in the mammary gland, and they play a crucial role in breast immunity (9–11). Neutrophils are activated in response to cytokines, pathogens, or specific compounds. Once activated, neutrophils capture and kill pathogens through phagocytosis, degranulation, and the release of neutrophil extracellular traps (NETs), which are extracellular web-like structures composed of decondensed chromatin and granular proteins. This process, known as NETosis, leads to the production of NETs (12). While NETs serve as a defensive mechanism against pathogens, their excessive formation and uncontrolled activation can lead to the destruction of normal tissue structures and sustained inflammation. Research on NETs has increased, and NETs are now considered biomarkers for many inflammatory and immune diseases (13). NETs represent potential targets for diagnosis and therapy. PAD4 is a catalytic enzyme that releases NETs by mediating histone citrullination. Inhibiting the activity of PAD4 reduces histone citrullination and chromatin decondensation, which in turn inhibits the formation of NETs (14, 15). Cl-amidine is a widely used inhibitor of PAD and has been demonstrated to reduce the inflammatory response in a fatal mouse model of sepsis (16, 17).

S100A8/A9 is a heterodimer composed of two proteins, S100A8 and S100A9. When released into the extracellular space, S100A8/S100A9 functions as a damage-associated molecular pattern (DAMP), activating inflammatory signaling pathways and promoting the production of inflammatory cytokines (18, 19). Furthermore, this signaling cascade results in increased expression of various ligands and receptors, creating a proinflammatory environment. The secretion of S100A8/S100A9 by immune cells establishes a positive proinflammatory feedback loop (20). Dysregulated S100A8/S100A9 expression has been linked to various inflammatory and autoimmune diseases (21, 22). Therefore, there has been growing interest in S100A8/S100A9 as a biomarker for monitoring inflammatory states and disease progression (23, 24).Previous studies have shown that neutrophils release S100A8/S100A9 through NETosis. This released S100A8/S100A9 subsequently activates neutrophils, thereby amplifying the response. The inhibition of S100A8/S100A9 may be a promising strategy for reducing the generation of NETs (18, 25).

Overall, further investigation into the relationship between autoimmunity and granulomatous lobular mastitis is crucial for the diagnosis and treatment of GLM. The present study aimed to provide a significant theoretical basis and explore clinical application prospects for the diagnosis and treatment of GLM.

## 2 Materials and methods

### 2.1 Human specimens

Surgical samples or peripheral blood samples were obtained from GLM patients and healthy controls (HCs) at our hospital. C57BL/6 female mice were used for the animal studies.

The present study included 40 patients diagnosed with granulomatous lobular mastitis in the GLM group and 20 healthy women without acute infections in the healthy control (HC) group. Fresh surgical specimens were collected from both groups and stored in liquid nitrogen for subsequent experiments.

The inclusion criteria for the GLM group were as follows: (1) a pathological diagnosis of granulomatous lobular mastitis, defined as a granulomatous inflammatory reaction centered on the lobules, with granulomas consisting of epithelioid histiocytes and Langhans giant cells, accompanied by infiltration of lymphocytes, macrophages, and neutrophils in and around the lobules; and (2) surgical treatment at our hospital. The exclusion criteria for the GLM group were as follows: (1) patients with other concurrent inflammatory diseases or malignant breast diseases and (2) patients who were pregnant or lactating.

The inclusion criteria for the HC group were patients who underwent surgical treatment for benign breast disease at our hospital at the same time and who were age matched. The exclusion criteria for the HC group were patients with concurrent inflammatory diseases or those who were pregnant or lactating.

Peripheral blood samples were collected from 20 healthy control (HC) subjects for the isolation of neutrophils.

The present study was conducted in accordance with the Declaration of Helsinki (as revised in 2013), and it was approved by the Ethics Committee of Nanjing Drum Tower Hospital (No. 2023-460-02). Informed consent was obtained from all patients.

### 2.2 Animal model

Female BALB/c mice (aged 6 to 8 months) with a history of pregnancy and lactation, were purchased from Beijing Charles River Laboratories (CRL). The mice weighed between 25 and 30 g and were housed under SPF conditions at the animal center. The mice were house under the following environmental parameters: temperature, 22–26°C; daily temperature variation, ≤4°C; relative humidity, 40–70%; ventilation rate, 15–20 times per hour; air cleanliness level, class 7; colony count, ≤3 per plate; ammonia concentration, ≤14 mg/m³; noise level, ≤60 dB; and light/dark cycle, 12/12 hours. The mouse husbandry process strictly followed guidelines for the care and use of laboratory animals.

#### 2.2.1 Experimental grouping

Validation of the GLM mouse model: Sixteen mice were randomly divided into the following two groups, with 8 mice per group: the control group and the GLM group. On the first day of model establishment, the GLM group was injected with a mixture of processed surgical specimen homogenate from granulomatous lobular mastitis and complete Freund’s adjuvant into the third and fourth pairs of mammary glands. The control group received an equal volume of saline mixed with complete Freund’s adjuvant at the same locations. The mice in each group were maintained under standard conditions after modeling.

**Figure 1:**
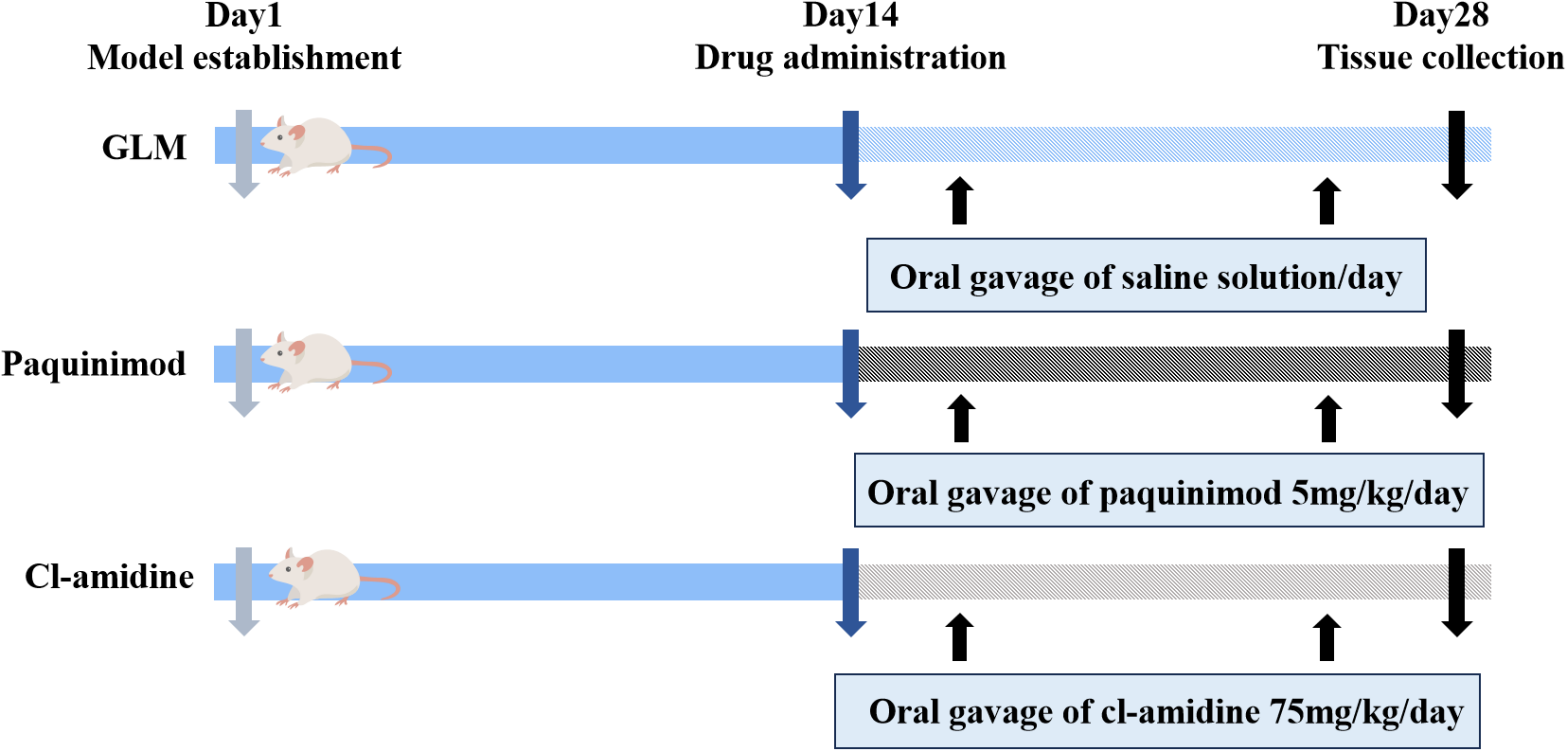
experimental grouping and model establishment schematic.

Evaluation of antagonist efficacy: Twenty-four mice were randomly divided into the following three groups, with 8 mice per group: GLM group, the paquinimod group, and the Cl-amidine group. On the first day, the GLM model was established for all groups. At two weeks post-modeling, antagonist interventions were administered after confirming successful model establishment. The paquinimod group received 5 mg/kg/day of paquinimod by gavage, and the Cl-amidine group received 75 mg/kg/day of Cl-amidine. For the control, the GLM group received an equal volume of saline by gavage (**Figure 1**).

#### 2.2.2 Specific steps for GLM model establishment

**Figure 2:**
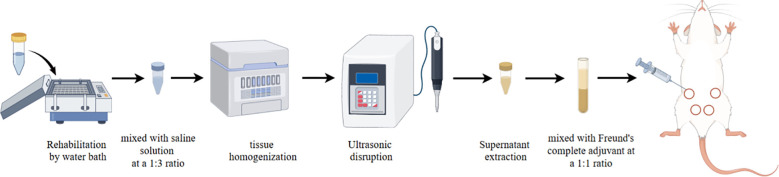
schematic diagram of GLM model establishment (drawn by Figdraw).

Under sterile conditions, fresh and uncontaminated pathological tissue from GLM patients was excised, placed in sterilized cryovials, transported to the laboratory and preserved in liquid nitrogen. Prior to modeling, the samples were thawed in a 40°C water bath for approximately 100 minutes. The pathological tissue was trimmed into small pieces, mixed with saline at a 1:3 ratio (1 g tissue to 3 ml saline), and transferred to a sterilized homogenization tube. (**Figure 2**) The mixture was homogenized at 3–4°C until completely ground. The homogenate was then treated with an ultrasonic cell disruptor (power, 120 W; intermittent mode, 10 seconds on and 3 seconds off for a total of 60 cycles). The supernatant was collected by centrifugation. The supernatant was mixed with complete Freund’s adjuvant (FCA) at a 1:1 ratio and then repeatedly mixed with a syringe for 20 minutes to form an oil-in-water emulsion for further experiments. After a 3-day acclimatization period at the animal center, BALB/c mice were anesthetized using 4% isoflurane. The skin was disinfected with iodine solution. After anesthesia, the mice were secured on a board, and the skin over the third and fourth mammary glands was disinfected with 75% ethanol. The nipples were grasped with forceps, and the inoculum was injected along the base of the nipple at an angle, with a depth of 2–4 mm; 0.04 ml was injected into each of the third and fourth mammary glands. Gentle pressure was applied after injection to achieve hemostasis.

#### 2.2.3 Specimen collection

Every 2 days, the transverse diameter and skin color of the mammary glands of the mice in each group were observed and recorded. At the end of the experiment, the mice in each group were euthanized, and the tissues from the third and fourth mammary glands were excised. After being washed with saline, a portion of the tissue was fixed in 4% paraformaldehyde, while another portion of the tissue was preserved in sterile cryovials and stored in liquid nitrogen for subsequent experiments.

### 2.3 Reagents and antibodies

#### 2.3.1 Reagents

A human blood neutrophil isolation kit (LZS11131) was obtained from Tianjin Haoyang Biotechnology Manufacture Co., Ltd. (Tianjin, China). A dsDNA quantification kit (12643ES) was obtained from Yeasen Biotechnology Co., Ltd. (Shanghai, China). Phorbol 12-myristate 13-acetate (PMA) and complete Freund’s adjuvant (CFA) were obtained from Beyotime Biotechnology Co., Ltd. (Shanghai, China). Cl-amidine was obtained from Selleck Chemicals (Shanghai, China). Paquinimod was obtained from MedChemExpress (MCE) (Shanghai, China). RPMI-1640 medium and fetal bovine serum (FBS) were obtained from Gibco (Thermo Fisher Scientific, USA). Phosphate-buffered saline (PBS), dimethyl sulfoxide (DMSO), Tween-20, sodium dodecyl sulfate (SDS), and all other general chemicals and buffers, unless otherwise specified, were obtained from Beijing Solarbio Science & Technology Co., Ltd. (China).

#### 2.3.2 Antibodies

The following antibodies were utilized in the present study:

anti-myeloperoxidase (MPO) (Abcam, ab208670); anti-citrullinated histone H3 (CitH3) (Abcam, ab281584); anti-S100A8/A9 (Abcam, ab288715); anti-beta actin (Abcam, ab8226); anti-citrullinated histone H3 (Cell Signaling Technology, #97272); anti-myeloperoxidase (MPO) (Proteintech, 66177-1-Ig); and anti-PAD4 (Proteintech, 17373-1-AP). According to the datasheets, all antibodies were confirmed to be reactive with human, mouse, and rat.

### 2.4 Experimental groups and dosing regimens *in vitro*

Neutrophils were seeded in a 24-well plate at a density of 2×10^5^ cells/ml in RPMI-1640 medium supplemented with 10% FBS. The final volume in each well was 500 µl. The cells were incubated in a humidified incubator at 37°C with 5% CO_2_ for the duration of the experiment. The experimental groups were designated as follows: group A (Control), the cells were treated with PBS and DMSO at the same concentrations as those used in the antagonist groups; group B (PMA), the cells were stimulated with PMA at a final concentration of 50 nM; group C (Cl-amidine + PMA), the cells were pretreated with Cl-amidine at a final concentration of 200 μM for 30 minutes, followed by the addition of PMA at a final concentration of 50 nM; and group D (paquinimod + PMA), the cells were pretreated with paquinimod at a final concentration of 100 μM for 30 minutes, followed by the addition of PMA at a final concentration of 50 nM. After 3 hours of treatment, the cells and supernatants were separated by centrifugation. The supernatant was collected for dsDNA fluorescence quantification analysis of extracellular NET release. The corresponding cell pellet was lysed for evaluation of intracellular protein expression and modification by Western blot analysis.

### 2.5 Experimental groups and dosing regimens *in vivo*

To verify the therapeutic effects of the paquinimod and Cl-amidine inhibitors on GLM, 24 mice were randomly divided into the following 3 groups, with 8 mice per group: the GLM group, the paquinimod group and the Cl-amidine group. On the first day, the GLM was established for all groups. After two weeks when successful modeling was confirmed, the inhibitors were administered. In the paquinimod group, the mice received a daily oral gavage of paquinimod at a dosage of 5 mg/kg/day. In the Cl-amidine group, the mice received a daily oral gavage of Cl-amidine at a dosage of 75 mg/kg/day. The GLM group served as the control group and received an equivalent volume of physiological saline daily by oral gavage.

During model establishment, changes in the diameter of the mouse mammary glands were recorded. After the completion of the experiment, the mice were anesthetized with 4% isoflurane, and mammary gland tissues were collected for subsequent experiments.

### 2.6 Sample collection and preservation

In total, 60 surgical specimens from the GLM and HC groups were collected from the pathology department, and sections were prepared for subsequent experiments. Fresh surgical specimens from both the GLM and HC groups were collected and preserved for later experiments. To minimize potential degradation of NETs, all the samples were processed immediately after collection. Tissue samples for protein analysis were snap-frozen in liquid nitrogen and stored at -80°C, while samples for histology were fixed immediately in 4% paraformaldehyde.

### 2.7 Isolation of human neutrophils

Peripheral blood from healthy volunteers was collected in EDTA-anticoagulated vacuum collection tubes for subsequent isolation of neutrophils. Neutrophils were isolated using a human blood neutrophil isolation kit according to the manufacturer’s instructions. The collected blood was separated within 2 hours of collection. In brief, the neutrophil cell separation reagent was added to a 15 ml sterile centrifuge tube, and the blood was carefully layered above the surface of the separation reagent. The samples were centrifuged for 30 min at room temperature at 600 g. After centrifugation, two distinct milky white layers were observed in the centrifuge tube; the upper layer consisted of mononuclear cells, and the lower layer consisted of neutrophils. The neutrophil layer was carefully pipetted into a new centrifuge tube, and 10 ml of wash solution was added. The samples were centrifuged for 10 min at 250 g, and the precipitate was collected. Erythrocyte lysate was added if the neutrophils were mixed with red blood cells. The cells were resuspended in 5 ml of wash solution, and the samples were centrifuged for 10 min at 250 g. After discarding the supernatant, the isolated neutrophils were resuspended in RPMI-1640 medium supplemented with 10% FBS and counted using a hemocytometer before being seeded for experiments.

### 2.8 Coimmunoprecipitation

Treated neutrophils were lysed using IP lysis buffer (25 mM Tris-HCl [pH 7.4], 150 mM NaCl, 1% NP-40, 1 mM EDTA, and 5% glycerol) supplemented with protease inhibitor cocktail (Roche, Switzerland). The cell lysate was then incubated with protein A/G agarose magnetic beads (MedChemExpress, China) conjugated with anti-PAD4 (Proteintech, 17373-1-AP) and anti-S100A8/A9 (Abcam, ab288715) antibodies overnight at 4°C. After being washed three times with lysis buffer, the bead-bound samples were subjected to Western blot analysis.

### 2.9 dsDNA assay

The cell culture supernatants were collected and centrifuged to remove cellular debris. The dsDNA concentration was measured using a dsDNA BR Assay Kit, (Yeasen, 12643ES) according to the manufacturer’s specifications. The fluorescence was measured using a microplate reader (BioTek, Synergy H1, USA).

### 2.10 Immunohistochemistry

Breast tissue samples removed during surgery were collected, fixed in 10% neutral buffered formalin, embedded in paraffin, and cut into 3 μm sections. The paraffin sections were deparaffinized in xylene and hydrated through a graded series of ethanol to tap water. Antigen retrieval was performed by microwaving the sections in sodium citrate buffer until the buffer started to boil. The sections were then allowed to stand for 15 min and cooled to room temperature. The sections were incubated in 3% hydrogen peroxide solution for 10 minutes at room temperature to quench endogenous peroxidase activity. After being rinsed three times with phosphate-buffered saline (PBS), the sections were incubated with the primary antibody overnight at 4°C. After three washes with PBS, the appropriate secondary antibody was added, followed by incubation at room temperature for 1 h. After being washed three times with PBS, the sections were developed with 3-3′-diaminobenzidine (DAB), counterstained with hematoxylin, dehydrated, cleared, and mounted with neutral balsam.

### 2.11 Immunofluorescence

Paraffin sections were prepared following the IHC protocol up to the antigen retrieval step when the sections were incubated with the appropriate primary antibody overnight at 4°C. After being rinsed three times with Tris-buffered saline with Tween-20 (TBST), the appropriate fluorescently labeled secondary antibody was added, followed by incubation at room temperature for 1 h in the dark. All subsequent steps were performed in the dark. After three TBST washes, the sections were mounted using anti-fluorescence quenching mounting medium (containing DAPI). Finally, coverslips were applied, and the sections were observed under a fluorescence microscope.

### 2.12 Hematoxylin and eosin staining

After the mice were sacrificed, their mammary tissues were collected and fixed in 10% neutral buffered formalin. The tissues were then dehydrated through a graded series of ethanol, cleared in xylene, and embedded in paraffin. Sections (3–5 μm) were cut and stained with hematoxylin and eosin using standard protocols.

### 2.13 Evaluation of immunohistochemical and immunofluorescence variables

Immunohistochemical and immunofluorescence results were assessed by 2 independent investigators. For each sample, four random fields of view per section were selected under low magnification. The percentage of positive staining area in these four fields was quantified using ImageJ software.

### 2.14 Immunoblotting

Cells were lysed in RIPA lysis buffer containing protease and phosphatase inhibitor cocktail. The protein concentration was determined using a BCA protein assay kit. Proteins (20–30 µg per lane) were separated on 10–15% SDS–PAGE gels and transferred onto PVDF membranes using a Mini-Trans Blot Electrophoretic Transfer System. The membranes were subsequently washed with TBST, blocked with 5% nonfat milk in Tris-buffered saline with 0.1% Tween-20 (TBST) for 1 h at room temperature, and incubated with primary antibodies in blocking solution at 4°C overnight. The membranes were then washed three times with TBST and incubated with horseradish peroxidase-conjugated secondary antibodies (1:5000) for 1 hour at room temperature. The protein bands were detected by ECL and visualized using a chemiluminescence imaging system.

### 2.15 Statistical analysis

The experimental results were analyzed using GraphPad Prism software version 9.0. The results are presented as the mean ± SEM. Student’s t test was used to compare the means of two independent groups with normally distributed data. The Mann–Whitney U test was used to compare variables of independent groups without normally distributed data. One-way ANOVA or Welch ANOVA was used for multiple groups with a normal distribution. The Kruskal–Wallis test was used for multiple groups without normally distributed data. A p value of less than 0.05 was considered to indicate statistical significance.

## 3 Results

### 3.1 NETs formation is enhanced in neutrophils from patients with GLM

**Figure 3:**
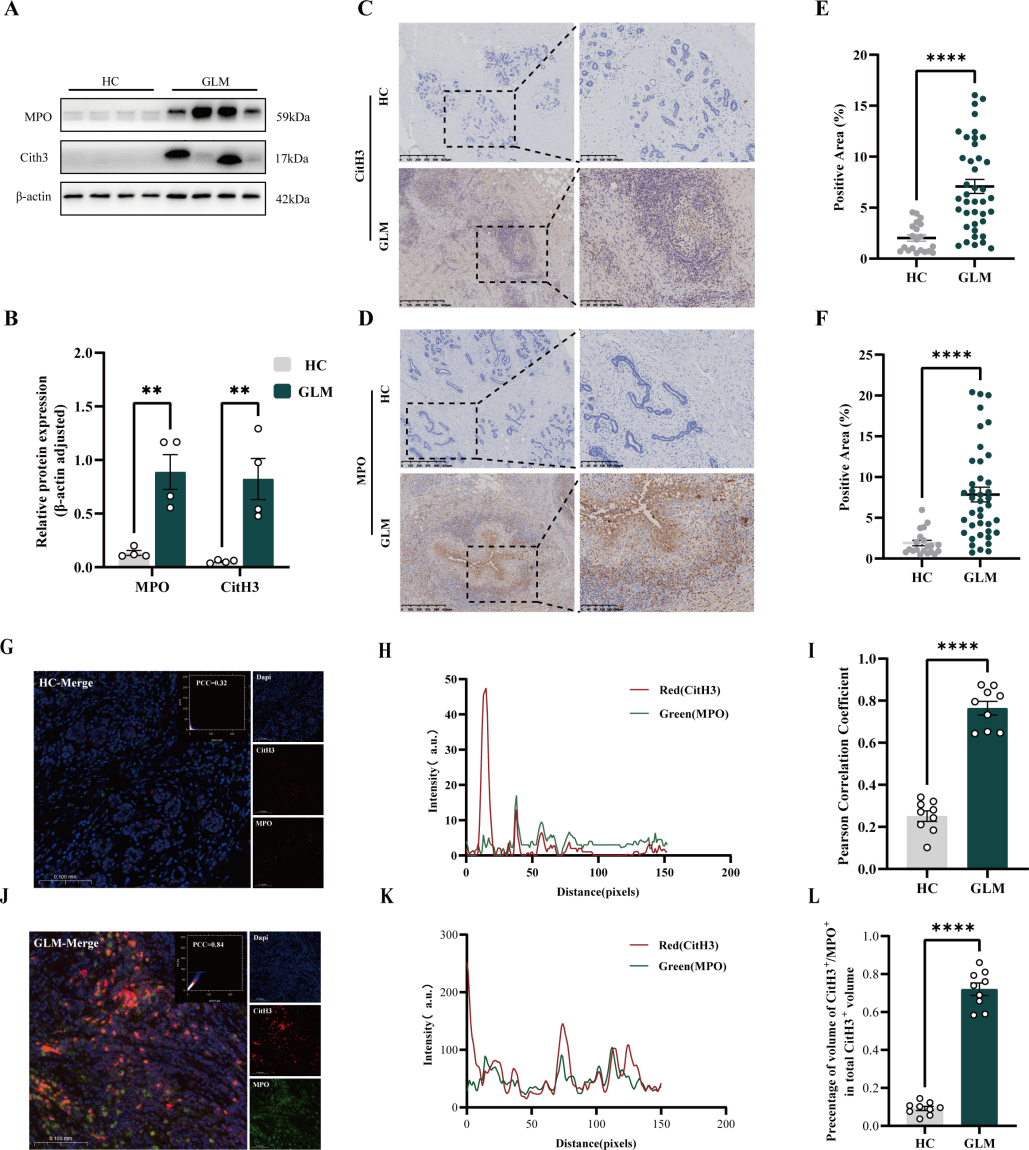
NETs expression in breast tissues in the GLM group and HC group. **(A)** Western blot results of NETs-related protein expression in the GLM and HC groups breast tissues, using β-actin as an internal reference. **(B)** The relative expression of CitH3 and MPO was quantified based on the gray values of the Western blot results (**p < 0.01). **(C)** Immunohistochemical staining results of CitH3. **(D)** Immunohistochemical staining results of MPO. **(E)** Analysis of the percentage of the positive area of CitH3 immunohistochemical staining between the two groups. (****p < 0.0001). **(F)** Analysis of the percentage of positive area of MPO immunohistochemical staining between the two groups. (****p < 0.0001). **(G, J)** The merged fluorescence images show the co-localization of CitH3 (red), Dapi (blue), and MPO (green). The scatter plots and Pearson’s correlation coefficient (PCC) indicating the fluorescence co-localization are shown in the images. **(H)** Distribution of fluorescence intensities in the HC group, where the red and green lines represent the fluorescence channels of CitH3 and MPO, respectively. **(K)** Distribution of fluorescence intensities in the GLM group. **(I)** Pearson’s correlation coefficient was used to evaluate the correlation between MPO (green fluorescence) and CitH3 (red fluorescence) in the HC group and the GLM group. **(L)** Quantitative analysis of the percentage of CitH3+/MPO+ co-localization area relative to the total CitH3+ area was performed. (****p < 0.0001).

To evaluate the presence of NETs in GLM patients, Western blot analysis was used to measure the levels of CitH3 and MPO in fresh samples from GLM patients and healthy controls (HCs). Citrulline histone H3 (CitH3) and myeloperoxidase (MPO) are specific markers of NETs. Compared with those in HCs, elevated levels of CitH3 and MPO were observed in the tissues of GLM patients, suggesting abundant NETs formation in GLM lesions (**Figures 3A–B**). Immunohistochemical staining was employed to compare the expression of these NET markers between GLM patients and HCs. In GLM tissues, a wide distribution of CitH3 and MPO staining was observed (**Figures 3C–F**), further verifying the elevated expression of the two NET-related proteins in GLM. Because NETs form a mesh-like structure, demonstrating NETs formation in GLM tissue samples is necessary. The colocalization of NETs components, specifically MPO and CitH3, in neutrophils suggested that NETs are abundantly formed in GLM (**Figures 3G–L**).

The spontaneous generation of numerous NETs in GLM lesions, even in the absence of specific external stimuli, implied that NETs are actively produced within the GLM microenvironment and are densely and abundantly distributed.

### 3.2 S100A8/S100A9 is highly expressed in GLM and may be a good predictor of GLM together with NETs proteins

**Figure 4:**
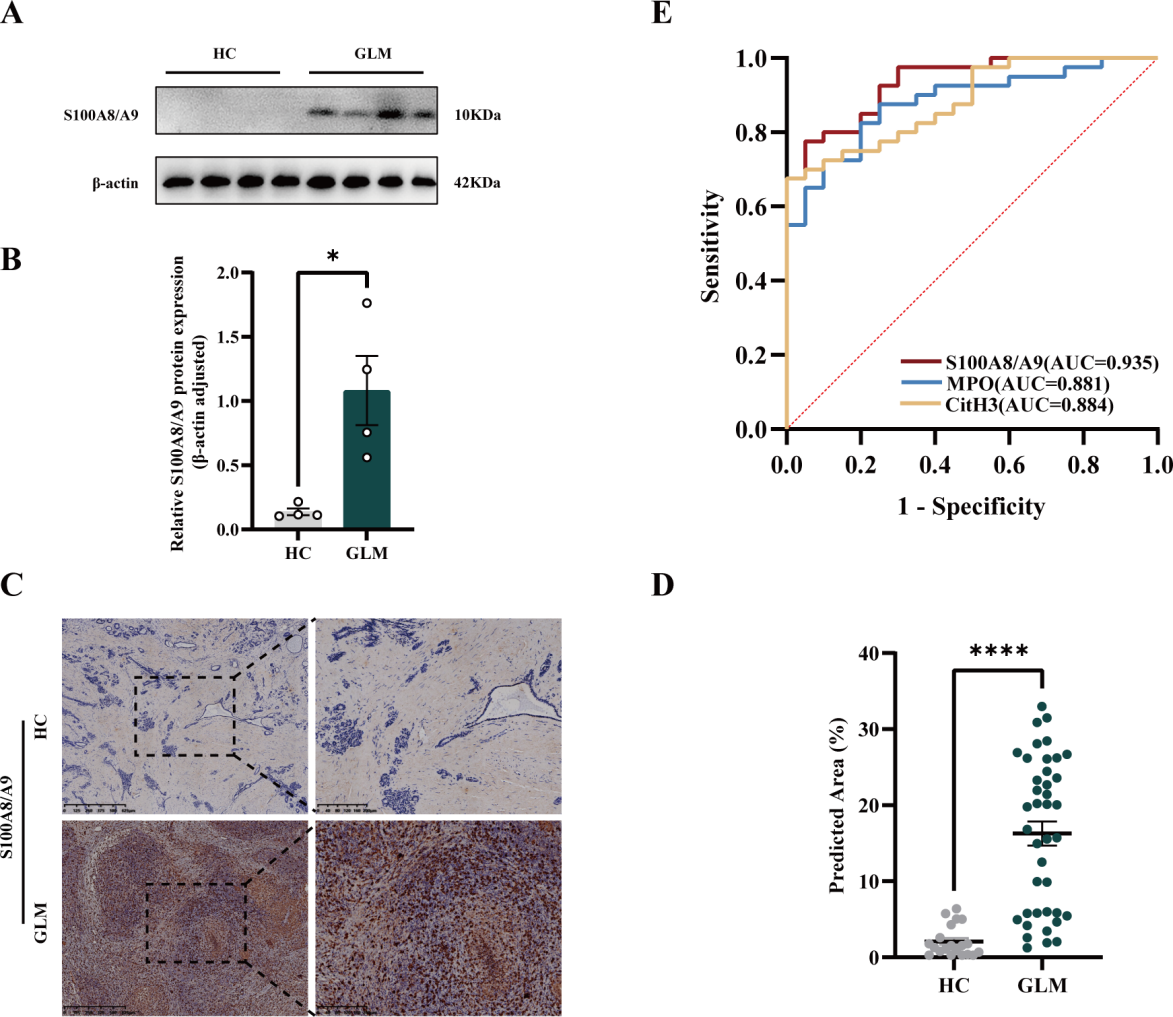
S100A8/A9 expression in breast tissues in the GLM group and HC group. **(A)** Western blot result plots with β-actin as an internal reference. **(B)** The relative expression of S100A8/S100A9 was quantified based on the gray values (*p < 0.05). **(C)** Graph of immunohistochemical staining results. **(D)** Analysis of the percentage of the positive area of S100A8/S100A9 immunohistochemical staining between the two groups (****p < 0.0001). **(E)** ROC curves of S100A8/S100A9, MPO, and CitH3 expression for the diagnosis of GLM.

GLM is a disease with an uncertain etiology and diagnostic challenges. To further explore the pathogenesis of GLM, S100A8/S100A9 was evaluated. S100A8 and S100A9 are proteins released by activated granulocytes during the formation of NETs, which further induce neutrophil activation and create a positive feedback loop, leading to prolonged inflammation, consistent with the persistent inflammation observed in GLM. Western blot and immunohistochemistry analyses of S100A8/S100A9 expression revealed that S100A8/S100A9 expression was elevated in the GLM group compared with the HC group (**Figures 4A–D**). To explore whether S100A8/S100A9, in combination with NETs proteins, may be a useful predictor of GLM, receiver operating characteristic (ROC) curves were generated by measuring the percentage of the positive area for the three proteins (**Figure 4E**). The expression levels of S100A8/S100A9, MPO, and CitH3 were significantly predictive of GLM.

### 3.3 S100A8/S100A9 facilitates NETs formation through a mechanism involving PAD4

**Figure 5:**
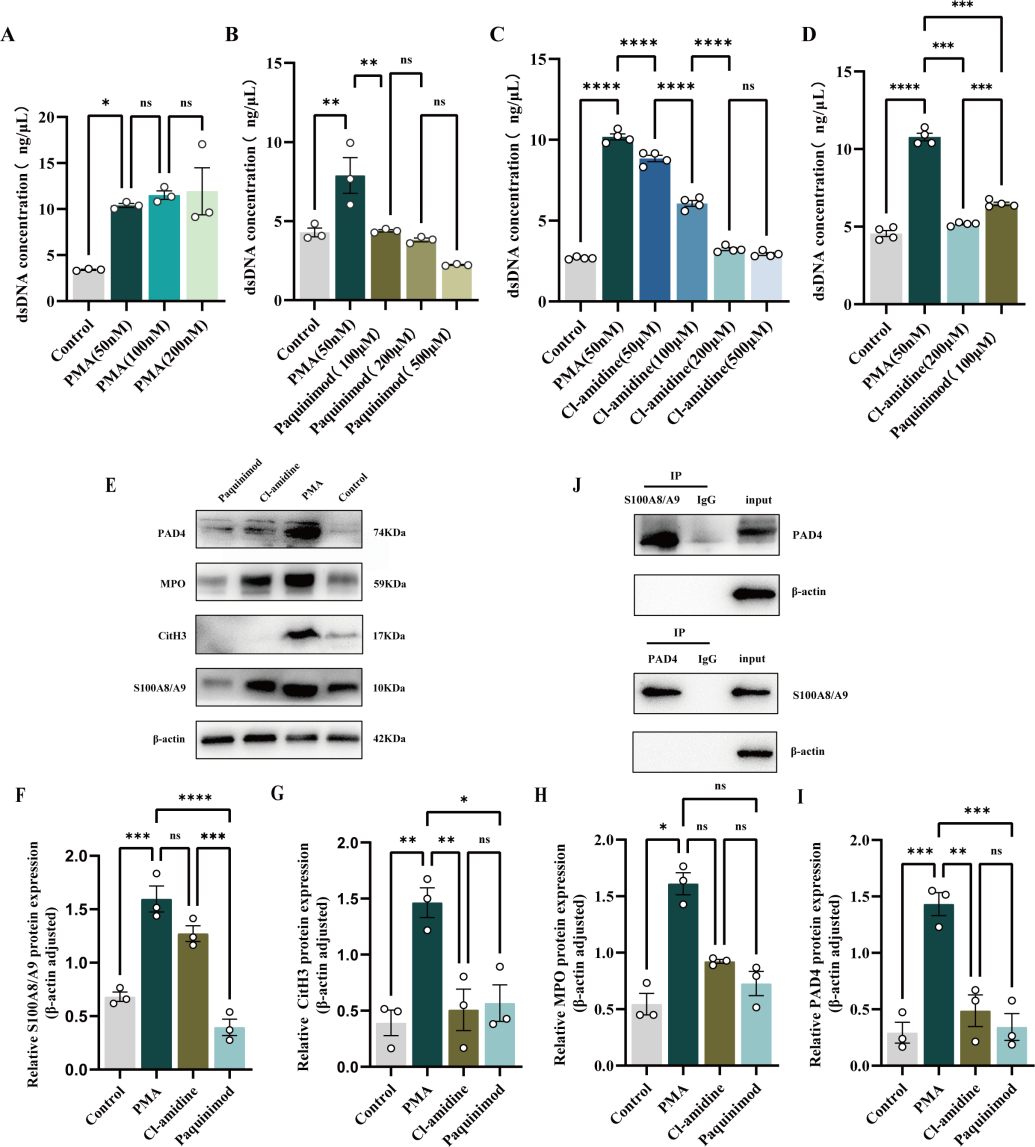
**(A)** PMA-induced formation of NETs in neutrophils (*p < 0.05). **(B)** Inhibitory effect of Paquinimod on NETs. Neutrophils were treated with 50, 100, 200, and 500 μM of Paquinimod, and dsDNA concentration was measured for evaluation (**p < 0.01). **(C)** Inhibitory effect of Cl-amidine on NETs. Neutrophils were treated with 50, 100, 200, and 500 μM of Cl-amidine, and the dsDNA concentration was measured for evaluation (****p < 0.0001). **(D)** Comparison of dsDNA production between Cl-amidine and Paquinimod. dsDNA concentration was measured to evaluate the inhibitory effects of the two antagonists (***p < 0.001, ****p < 0.0001). **(E)** Western blot results of NETs-related protein expression with β-actin as an internal reference. **(F–I)** The relative expression was quantified on the basis of the gray values (*p < 0.05, **p < 0.01, ***p < 0.001, ****p < 0.0001). **(J)** Co-IP experiments verifying the protein-protein interaction between S100A8/S100A9 and PAD4, showing that S100A8/S100A9 interacts with PAD4.

To investigate the role of S100A8/S100A9 in promoting NETs formation, we isolated primary human neutrophils from peripheral blood. A NETs generation model was established using phorbol 12-myristate 13-acetate (PMA), a known potent inducer of NETosis. Compared with that in the control group, quantitative fluorescence analysis of dsDNA in the supernatant confirmed that PMA stimulation significantly increased NETs release (**Figure 5A**).

To further elucidate this pathway, specific inhibitors, namely, paquinimod (an S100A8/S100A9 inhibitor) and Cl-amidine (a PAD4 inhibitor), were applied 30 minutes prior to PMA stimulation. We added paquinimod or Cl-amidine 30 minutes before adding PMA. After PMA treatment, the cells were incubated at 37°C for 3 hours, and the supernatant was centrifuged for quantitative fluorescence analysis of dsDNA. Compared with the control, treatment with 100 μM paquinimod or 200 μM Cl-amidine significantly inhibited dsDNA release (**Figures 5B–D**). Western blot analysis revealed that paquinimod treatment markedly inhibited the expression of S100A8/S100A9, CitH3, and PAD4, whereas Cl-amidine treatment markedly inhibited the expression of PAD4 and CitH3 but not S100A8/S100A9 (**Figures 5E–I**). These findings suggested that S100A8/S100A9 is upstream of PAD4 activation in the NETosis pathway.

To determine whether S100A8/S100A9 itself is sufficient to trigger NETosis, unstimulated neutrophils were treated with the purified S100A8/S100A9 heterodimer, which induced NET formation comparable to that induced by PMA, as measured by dsDNA release (**Supplementary Figure S1A**). These data collectively indicated that S100A8/S100A9 acts as a marker and a potent upstream trigger of NETosis. Consistently, Western blot analysis revealed a concurrent, dose-dependent upregulation of key NETosis-related proteins, including PAD4 and its product, namely, citrullinated histone H3 (CitH3) (**Supplementary Figures S1B-F**).

To determine if the promotion of NETs by S100A8/S100A9 involves direct functional interplay with PAD4, coimmunoprecipitation (Co-IP) experiments were performed, which revealed that the interaction between S100A8/S100A9 and PAD4 was strictly dependent on cellular activation. This complex was detected in PMA-stimulated neutrophils but was absent in unstimulated, resting neutrophils (**Figure 5J**, **Supplementary Figure S1G**). This activation-dependent association suggested that the formation of the S100A8/S100A9-PAD4 complex is a specific event in the NETotic process.

To rule out the possibility that this interaction is a passive consequence of both proteins being coentrapped within the extruded chromatin meshwork of NETs, lysates from activated neutrophils were treated with DNase I prior to Co-IP. The interaction between S100A8/S100A9 and PAD4 persisted after enzymatic degradation of the DNA backbone (**Supplementary Figure S1H**). These findings demonstrated that the interaction between S100A8/S100A9 and PAD4 is direct or mediated by a stable protein complex and is not an artifact of NET entrapment.

In summary, the present results demonstrated that S100A8/S100A9 is both a potent inducer and a key regulator of NET formation. This process is mechanistically linked to a specific, activation-dependent interaction with PAD4 that is critical for promoting NETosis, which occurs independently of DNA entrapment within NETs.

### 3.4 Targeting NETs can effectively inhibit GLM production

**Figure 6:**
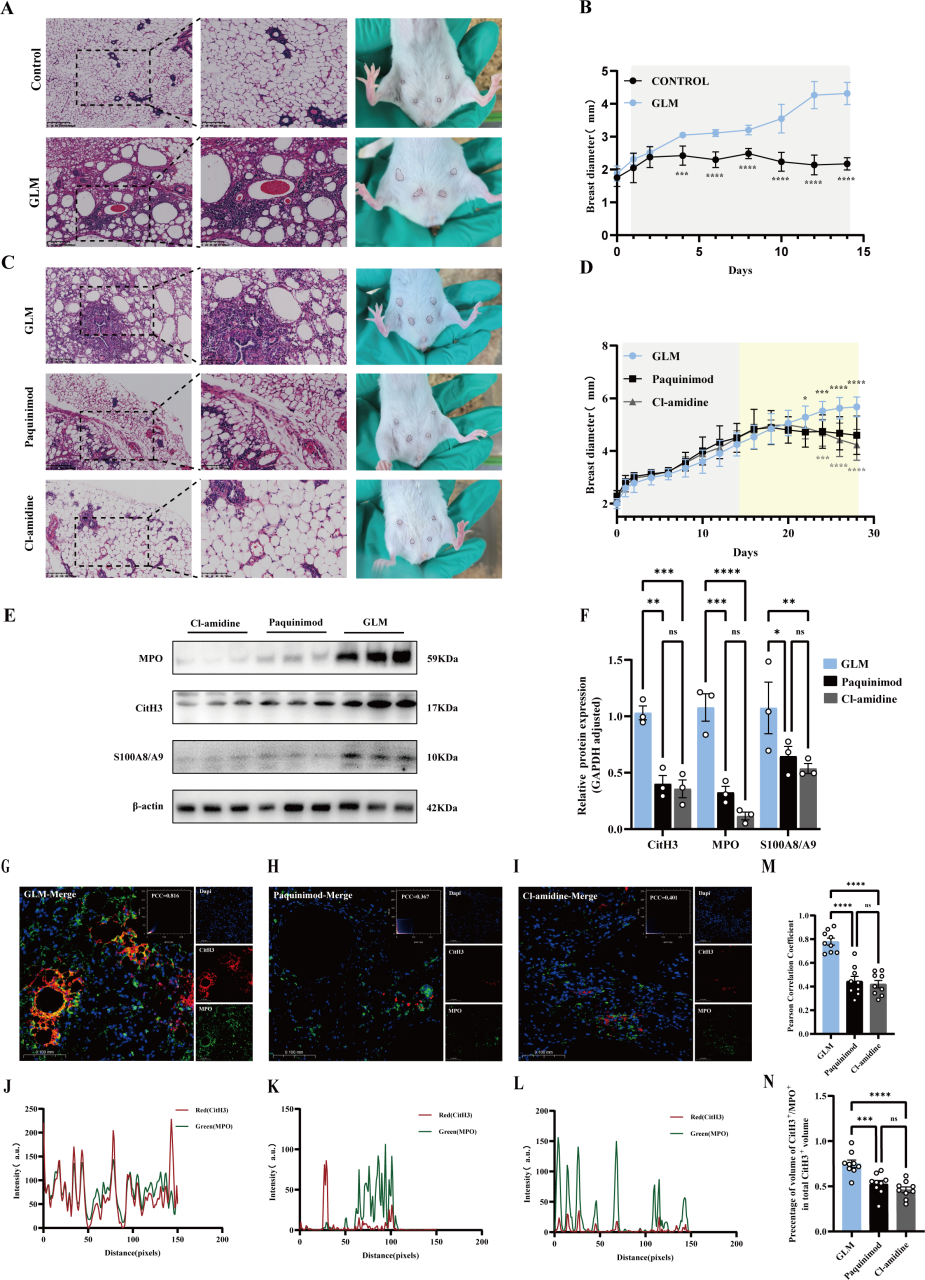
**(A)** H&E staining: H&E staining of mammary glands in the control group mice showed a normal mammary gland structure, with cell nuclei stained dark blue or purple, and cytoplasm stained pink or red. The boundary of mammary lobular tissue was clear. H&E staining of mammary glands in the GLM group mice showed unclear boundaries of mammary lobular tissue, with extensive infiltration of lipid vacuoles, lymphocytes, and neutrophils. **(B)** Changes in mammary gland diameter in both groups of mice, with the mammary gland diameter of the GLM group mice larger than that of the control group mice (****p < 0.0001). **(C)** H&E staining: H&E staining of mammary glands in GLM group mice showed unclear boundaries of mammary lobular tissue, with extensive infiltration of lipid vacuoles, lymphocytes, and neutrophils. H&E staining of mammary glands in the Cl-amidine and Paquinimod groups showed reduced inflammatory cell infiltration compared to the GLM group. **(D)** Changes in mammary gland diameter in the three groups of mice, with the mammary gland diameter of GLM group mice larger than that of the Cl-amidine and Paquinimod groups. No significant difference in mammary gland diameter was observed between the Cl-amidine and Paquinimod groups (***p < 0.001, ****p < 0.0001). **(E)** Western blot results of NETs-related protein expression with β-actin as an internal reference. **(F)** The relative expression was quantified based on the gray values (*p < 0.05, **p < 0.01, ***p < 0.001, ****p < 0.0001). **(G–I)** Fluorescence co-localization images of NETs-related proteins in the three groups, with the three overlapping fluorescence channels representing CitH3 (red), DAPI (blue), and MPO (green). The scatter plots and Pearson’s correlation coefficient (PCC) of fluorescence co-localization are annotated in the images. **(J–L)** Distribution of fluorescence intensities in the three groups, with the red and green lines representing the fluorescence channels of CitH3 (red) and MPO (green), respectively. **(M)** Evaluation of the correlation between MPO (green fluorescence) and CitH3 (red fluorescence) among the three groups using Pearson’s correlation coefficient. **(N)** Quantitative analysis of the percentage of CitH3+/MPO+ co-localization area relative to the total CitH3+ area (***p < 0.001, ****p < 0.0001).

After determining that S100A8/S100A9 promotes NETs, we constructed a GLM mouse model. HE staining was used to confirm the successful establishment of the model, which revealed unclear boundaries of the lobular tissue in the breast, a large number of lipid vacuoles, and significant infiltration of lymphocytes and neutrophils, consistent with the pathological changes in GLM, indicating that the model was successfully constructed (**Figures 6A–C**). The mice were then treated with Cl-amidine and paquinimod by gavage. Compared with that in the GLM group, the mammary gland diameter in the inhibitor-treated groups tended to decrease over time (**Figure 6D**). Analysis of the expression levels of related proteins revealed that the inhibitors reduced the levels of MPO, CitH3, and S100A8/S100A9 (**Figures 6E, F**). Fluorescence colocalization analysis of NETs revealed that the Cl-amidine and paquinimod groups were significantly different from the GLM group, further verifying that blocking the generation of NETs effectively inhibits GLM pathology (**Figures 6G–N**). Taken together, these results indicated that paquinimod and Cl-amidine are effective at inhibiting NET formation and may represent novel and practical therapeutic strategies for GLM.

## 4 Discussion

The prevalence of GLM is relatively high prevalence in certain countries, including Turkey, Egypt, Iran, Pakistan, China, and Saudi Arabia (26). In some of these countries, GLM is linked to typical symptoms and manifestations of rheumatic diseases, such as arthritis and erythema nodosum (27). Additionally, certain HLA antigens are expressed at higher levels in some patients with GLM. These findings suggest that autoimmunity and immune dysregulation may contribute to the development of GLM.

Neutrophils play a crucial role in the immune system to regulate inflammation (28). When an infection occurs, neutrophils are activated and migrate to the site of infection or injury. Neutrophils act against infection through various functions, such as phagocytosis, granule release, and NETs formation (29). Activated neutrophils can adhere to the vascular endothelium and tissue cells, promoting neutrophil aggregation through interactions between adhesion molecules and damaged tissues. Upon aggregation, neutrophils release proteases, oxygen free radicals, and cytokines, which may increase the inflammatory response and trigger tissue damage (30). Numerous studies have investigated the therapeutic effects of small-molecule inhibitors or knockout mouse models against NETs. PAD4, one of the primary targets, plays a crucial role in neutrophil activation, NET formation, and the immune response by converting histone amino acid residues to citrulline, promoting chromatin remodeling (16, 31). PAD4 inhibitors, such as CI-amidine, have been shown to reduce disease severity in models of sepsis, arthritis, and ulcerative colitis (15, 31). Notably, S100A8 and S100A9, members of the S100 family, are particularly abundant in neutrophils (24). Together, they account for 40% of the cytoplasmic protein content of neutrophils and are released by activated granulocytes (21, 23).S100A8/A9 proteins bind to cell surface receptors in a cytokine-like manner, triggering signaling pathways involved in the inflammatory process (32). Abnormal expression and aberrant function of S100A8/S100A9 are closely associated with the onset and progression of a wide range of diseases, including inflammatory diseases, tumors, and autoimmune diseases (25, 33). Additionally, S100A8/S100A9 can activate neutrophils, induce their activation, and participate in key steps of NETosis (18, 19, 34). Paquinimod is an orally administered immunomodulatory compound that has been evaluated in clinical trials for its efficacy in treating various autoimmune and inflammatory diseases (35, 36). Paquinimod effectively inhibits S100A8/S100A9 activity, reducing inflammation. The protective effects of paquinimod have been demonstrated in mouse models of infection, autoimmune-induced neuroinflammation, and depression (37, 38).

The objective of the present study was to investigate the association between neutrophil activation and GLM. Additionally, we aimed to determine whether inhibition of NETs produced by neutrophil activation could reduce the degree of inflammation in GLM. We also examined the potential of Paquinimod as a novel NETs inhibitor. The expression of NETs and S100A8/S100A9 in the breast tissues of GLM patients was examined using WB, IHC and IF analyses. The presence of the CitH3 and MPO NETs-associated proteins CitH3 and MPO was detected in the breast tissues of patients with GLM. Moreover, the expression level of S100A8/S100A9 was significantly greater in patients with GLM than that in the controls. These results suggested that neutrophils activate and release NETs during the pathological process of GLM while also increasing S100A8/S100A9 expression. Methods commonly used for detecting NETs include flow cytometry, live-cell imaging, enzyme-linked immunosorbent assay (ELISA), IF confocal microscopy, and WB analysis (39). As there are no antibodies that specifically detect NETs, the identification of NETs is best achieved by detecting the colocalization of components (40), and immunofluorescence confocal microscopy is a commonly used method for visualizing and quantifying NETs. Therefore, we used previously preserved surgical specimens to detect the expression of NETs. Surgical specimens were collected from patient peripheral blood for dsDNA analysis to detect the expression of NETs. This decision was made because of the difficulty in obtaining a large number of patient peripheral blood samples simultaneously and the desire to visualize the presence and distribution of NETs through fluorescence confocal imaging. Owing to the limited number of surgical specimens, expanding the sample size in subsequent studies may improve the representativeness and reliability of the present results. Despite these limitations, the preserved surgical specimens provided valuable information regarding the relationship between GLM and neutrophil activation through the detection of NETs.

Neutrophils were extracted from human peripheral blood and activated with PMA to generate NETs. The dsDNA and Western blot analysis results indicated that both Cl-amidine and paquinimod significantly inhibited the release of NETs from neutrophils. These findings suggested that Cl-amidine and paquinimod act as antagonists of NET formation and have potential inhibitory effects. Additionally, treatment with Cl-amidine significantly decreased the expression levels of PAD4 and histone H3, which are proteins associated with the formation of NETs. These findings suggested that the mechanism by which Cl-amidine inhibits NET formation may involve the inhibition of PAD4 activity and chromatin untangling. These findings further support the inhibitory effect of Cl-amidine, a PAD4 inhibitor, on NET formation. In the present study, paquinimod effectively inhibited NET release from neutrophils. Paquinimod may have potential therapeutic applications as an S100A8/S100A9 inhibitor, as excessive release of S100A8/S100A9 is associated with the occurrence and development of various inflammatory and autoimmune disorders. By interfering with the function of S100A8/S100A9 and reducing the formation of NETs, paquinimod may modulate the inflammatory response and provide new therapeutic avenues and drug candidates for the treatment of related diseases. Co-IP confirmed the interaction between S100A8/S100A9 and PAD4, revealing that S100A8/S100A9 may regulate the activity and function of PAD4 through binding to PAD4, which in turn affects the formation of NETs. However, further research is needed to fully understand the mechanism underlying the interaction between S100A8/S100A9 and PAD4.

While the present *in vitro* models, including the use of PMA, are valuable for delineating the NETosis pathway, we acknowledge that such potent, nonphysiological stimuli do not fully replicate the complex inflammatory microenvironment of GLM. However, the identification of S100A8/S100A9 as a crucial upstream activator helps bridge this gap. As an endogenous DAMP abundantly expressed in GLM tissues, S100A8/S100A9 represents a physiologically relevant trigger. The present findings demonstrated that exogenous S100A8/S100A9 alone is sufficient to induce robust NETosis, thus supporting a plausible model in which local S100A8/S100A9 release initiates and propagates a PAD4-dependent NETosis feedback loop *in vivo*.

We also investigated the effect of inhibiting NET production on inflammatory damage in mammary tissue by treating GLM mice with two antagonists, namely, Cl-amidine and paquinimod. HE staining revealed that the mammary tissues of control mice exhibited significant inflammatory responses, such as inflammatory cell infiltration, disorganized and unclear mammary lobules, and lipid vacuole formation. In mice treated with Cl-amidine and paquinimod, however, the inflammatory response of mammary tissue was significantly reduced, and the tissue structure was protected. These findings suggested that Cl-amidine and paquinimod effectively reduce inflammatory damage in mammary tissue. Further WB analyses supported these observations. Compared with the GLM group, the antagonist group showed significant reductions in the expression of the CitH3, MPO, and S100A8/S100A9 NET-related proteins. The formation of NETs was also examined through immunofluorescence colocalization. Significant accumulation of NETs was observed in the mammary tissues of control mice, as evidenced by the colocalization of NET-associated proteins. The formation of NETs was significantly reduced in mice treated with Cl-amidine and paquinimod, indicating the effectiveness of these antagonists in inhibiting NET production. Moreover, no significant differences were observed between the Cl-amidine and paquinimod groups, suggesting that the two antagonists may have similar effects on the inhibition of NET generation. These results indicated that treatment of GLM model mice with Cl-amidine or paquinimod effectively reduces inflammatory damage in mammary tissue, possibly through the inhibition of NET production.

The present study had several limitations. First, the translatability of findings from murine models to human pathology may be constrained by interspecies differences in neutrophil biology. Compared with human neutrophils, which may utilize additional PAD4-independent pathways, murine neutrophils exhibit a stronger dependence on PAD4 for NET formation (41, 42). Although this difference may influence the relative efficacy of PAD4 inhibitors, such as Cl-amidine, across species, several aspects of the present study help to bridge this translational gap. In the present study, the initial observations of NETs and S100A8/S100A9 were derived directly from human GLM tissues, and the inhibitory effect of paquinimod was consistent in both human neutrophils *in vitro* and the mouse model. These convergent findings strengthen the relevance of the S100A8/S100A9-PAD4 axis in GLM.

Because the present findings highlight the therapeutic potential of targeting the S100A8/S100A9 axis in GLM, it is crucial to consider the potential consequences of its chronic inhibition on innate immunity and host defense. S100A8/S100A9 plays a role in modulating neutrophil recruitment and function, and it contributes to the antimicrobial activity of NETs (43, 44). Therefore, systemic and long-term suppression of S100A8/S100A9 may increase susceptibility to infections. However, several factors may mitigate this concern in the context of GLM management. First, GLM is a localized inflammatory condition, and future therapeutic approaches could explore localized drug delivery to minimize systemic exposure. Second, the clinical development of paquinimod in other chronic inflammatory diseases has provided some safety data. While an increased incidence of mild to moderate infections has been observed in some clinical trials, these infections are often manageable (45, 46). Thus, the immunosuppressive effect may be partial and context dependent. Finally, the treatment regimen for GLM may not necessitate perpetual inhibition but rather a finite course to break the cycle of chronic inflammation. Nonetheless, the present results underscore the importance of future clinical studies carefully monitoring for signs of infection and evaluating the risk–benefit ratio of S100A8/S100A9 inhibition in patients with GLM.

The present study had several methodological and mechanistic limitations. The present *in vitro* model relied on PMA as a standardized NETosis inducer, which does not fully replicate the pathological GLM microenvironment (47, 48). Future studies should employ more disease-relevant stimuli (such as Corynebacterium species) or cytokines (such as IL-8 and TNF-α). In addition, while the interaction between S100A8/S100A9 and PAD4 was confirmed, the precise mechanism of action of paquinimod and the functional significance of this interaction remain to be fully elucidated. A key question is whether S100A8/S100A9 promotes NETosis primarily through a direct interaction with PAD4 or via its canonical receptors. As a well-characterized damage-associated molecular pattern (DAMP), S100A8/S100A9 signals through receptors, such as Toll-like receptor 4 (TLR4) and the receptor for advanced glycation end products (RAGE) (40, 49). Activation of these receptors triggers potent proinflammatory signaling cascades, including the NF-κB and MAPK pathways, which modulate neutrophil activation and NETosis (50). Therefore, determining whether the inhibition of NETosis by paquinimod occurs solely through disruption of the S100A8/S100A9-PAD4 complex or through interference with these broader proinflammatory signaling pathways is crucial. Future studies should investigate the contribution of TLR4 and RAGE to NET formation in GLM to provide a more comprehensive understanding of the regulatory network and identify potential nodes for therapeutic intervention.

## 5 Conclusion

The present findings suggested that large numbers of NETs are produced in GLM and that these NETs cause damage to breast tissue. S100A8/S100A9 may be a key factor in the formation of NETs in GLM. GLM can be effectively detected by measuring the levels of S100A8/S100A9, CitH3, and MPO. In addition, paquinimod inhibits the formation of NETs by regulating S100A8/S100A9. Thus, S100A8/S100A9 may be a promising intervention target for inhibiting NETs in granulomatous lobular mastitis, which may be related to its ability to bind PAD4, suggesting the potential for application in the treatment of other inflammatory diseases.

## Data Availability

The original contributions presented in the study are included in the article/**Supplementary Material**. Further inquiries can be directed to the corresponding author.

## Supplementary material

Supplementary Figure 1 **(A)** Induction of NET formation by exogenous S100A8/S100A9 in neutrophils. (***p < 0.001, ****p < 0.0001). **(B)** Western blot results of NETs-related protein expression with β-actin as an internal reference. **(C–F)** The relative expression was quantified based on the gray values (*p < 0.05, **p < 0.01, ***p < 0.001, ****p < 0.0001). **(G)** Co-IP analysis of the interaction between S100A8/S100A9 and PAD4 in resting and PMA-activated neutrophils. **(H)** Co-IP analysis of the S100A8/S100A9 and PAD4 interaction in PMA-activated neutrophils following DNase I pretreatment of cell lysates.
