# Supplementary figures and images for: S100A8/S100A9 through PAD4 activation of neutrophil extracellular traps promotes granulomatous lobular mastitis
Source: Front Immunol. 2025 Dec 16;16:1672538. doi: 10.3389/fimmu.2025.1672538 (PMC12748195; doi:10.3389/fimmu.2025.1672538)

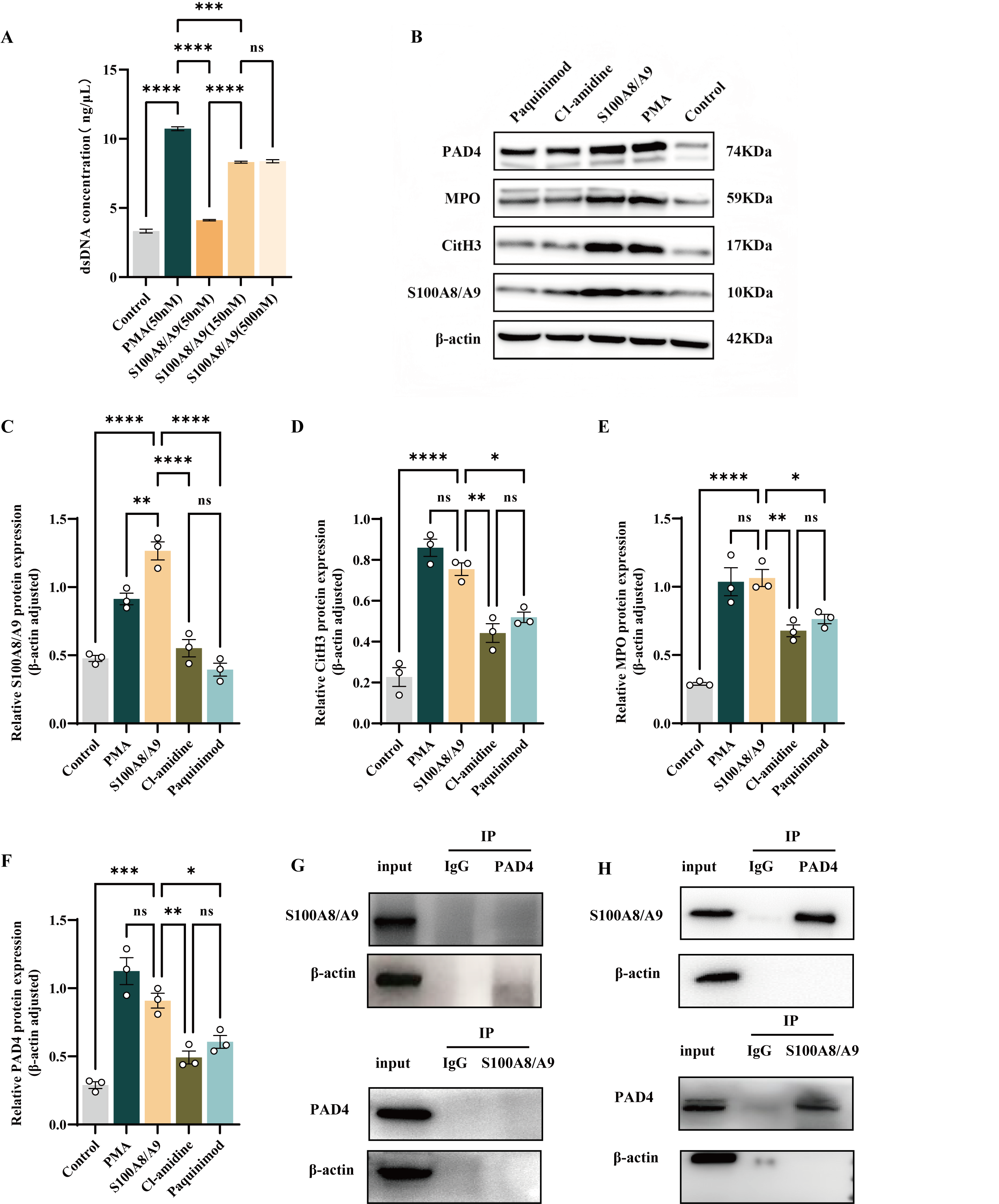

Supplement: Supplementary Figure 1 — (A) Induction of NET formation by exogenous S100A8/S100A9 in neutrophils. (***p < 0.001, ****p < 0.0001). (B) Western blot results of NETs-related protein expression with β-actin as an internal reference. (C–F) The relative expression was quantified based on the gray values (*p < 0.05, **p < 0.01, ***p < 0.001, ****p < 0.0001). (G) Co-IP analysis of the interaction between S100A8/S100A9 and PAD4 in resting and PMA-activated neutrophils. (H) Co-IP analysis of the S100A8/S100A9 and PAD4 interaction in PMA-activated neutrophils following DNase I pretreatment of cell lysates. [file Image1.tif]
